# Age-Related Changes of Elastic Fibers in Shoulder Capsule of Patients with Glenohumeral Instability: A Pilot Study

**DOI:** 10.1155/2018/8961805

**Published:** 2018-07-18

**Authors:** A. Castagna, E. Cesari, A. Gigante, B. Di Matteo, R. Garofalo, G. Porcellini

**Affiliations:** ^1^Center for Shoulder and Elbow Surgery, Humanitas Clinical and Research Center, Rozzano, Milan, Italy; ^2^Shoulder and Elbow Surgery Unit, Humanitas Gavazzeni Institute, Bergamo, Italy; ^3^Clinical Orthopaedics, Department of Clinical and Molecular Science, School of Medicine, Università Politecnica delle Marche, Ancona, Italy; ^4^Humanitas University, Department of Biomedical Sciences, Via Manzoni 113 and Humanitas Clinical and Research Center, Via Manzoni 56, 20089 Rozzano, Milan, Italy; ^5^Ospedale Generale Regionale “F. Miulli”, Acquaviva delle Fonti, Bari, Italy; ^6^Shoulder and Elbow Unit, “D. Cervesi” Hospital, Cattolica-AUSL della Romagna, Ambito Rimini, Italy

## Abstract

**Background:**

Recurrent shoulder dislocations occur much more frequently in adolescents than in the older population but a clear explanation of this incidence does not exist. The aim of the present study was to define the age-related distribution of the elastic fibers (EFs) in the shoulder capsule's extracellular matrix as a factor influencing shoulder instability.

**Materials and Methods:**

Biopsy specimens were obtained from the shoulder capsule of patients divided preoperatively into three groups: Group 1 consisted of 10 male patients undergoing surgery for unidirectional traumatic anterior instability (TUBS); Group 2 consisted of 10 male patients undergoing surgery for multidirectional instability (MDI); Group 3 represents the control, including 10 patients with no history of instability. In addition to the group as a whole, specific subgroups were analyzed separately on the basis of the age of subjects: > 22 or < to 22 years. All the samples were analyzed by histochemical (Weigert's resorcinol fuchsin and Verhoeff's iron hematoxylin), immunohistochemical (monoclonal antielastin antibody), and histomorphometric methods.

**Results:**

Both the elastin density and the percentage of area covered by EFs were significantly higher in younger subjects (<22 years old). Furthermore, the elastin density and the percentage of area covered by EFs were significantly higher in specimens of group of patients affected by multidirectional shoulder instability in comparison to the other two groups.

**Conclusion:**

Data of the present study confirmed the presence of an age-related distribution of EFs in the human shoulder capsule. The greater amount of EFs observed in younger subjects and in unstable shoulders could play an important role in predisposing the joint to first dislocation and recurrence.

## 1. Introduction

Glenohumeral instability is usually defined as a clinical syndrome characterized by shoulder pain related to abnormal displacement of the humeral head in the glenoid [1]. It represents a wide spectrum of pathologies and can be classified according to the timing of diagnosis and frequency of the event, the degree, the direction(s), and the etiology of the first occurrence (traumatic or atraumatic). With respect to direction, glenohumeral instability may be anterior, posterior, inferior, or multidirectional [1, 2]. Several authors showed the importance of distinguishing traumatic unidirectional instability (TUBS) from atraumatic multidirectional instability (AMBRI) [3–7].

In the pathogenesis of unidirectional recurrent shoulder instability, several factors have a recognised role: age under 22 years at time of trauma, male sex, involvement in competitive or contact sport, large Hill Sachs lesion, large Bankart or bony Bankart lesion, rotator cuff and biceps deficiency, glenohumeral dysplasia and abnormal version, and scapulothoracic dyskinesia [1, 2]. About the role of age, higher incidence of shoulder instability has been reported at the second and third decade and different authors showed that recurrent dislocation occurs much more frequently in adolescents than in the older population but a clear explanation of this incidence does not exist [8–11]. These studies just noted that age is one of the major risk factors for primary shoulder dislocation and recurrence [9]. Alterations of the capsulo-ligamentous structures are also commonly considered important predisposing factor to first dislocation and recurrence [1, 9], but the biological nature of these alterations has not been well investigated yet. They include constitutional glenohumeral laxity and acquired capsule alterations, which consist in a plastic deformation of the capsule and the inferior glenohumeral ligament complex that can occur also with a single anterior dislocation, even though tissue elongation is difficult to document both at MRI and intraoperatively [4, 12].

On the other hand, the pathogenesis of multidirectional instability is still not clear. It may be multifactorial, but capsular laxity (i.e., a loose, redundant inferior capsule) is a pathological predisposing condition that has been implicated as one of the main pathogenetic factors. The entity of laxity is related to age, sex, and genetic factors which control the histological structure and biochemical composition of articular capsule [1, 13–15].

The glenohumeral articular capsule is a dense fibrous connective tissue composed mainly of water, proteoglycans, collagen, and elastic fibers [16]. Collagen fibril diameter has been shown to be correlated with the tensile strength of shoulder capsule [17]. The elastic fibers (EFs) are one of the main components of the connective tissue that provide physiologic elasticity to it, and may affect capsular strength [18]. Abnormalities in EFs and specifically in the fibrillin component have been demonstrated in Marfan's syndrome, a condition that is associated with connective tissue laxity [19, 20]. The distribution of elastic fibers is related to the different functional role and biomechanical behaviour of each tissue and the amount of elastic fibers in the various tissues changes with age [18, 21, 22]. Very few studies have analyzed the histological and biochemical composition of the human shoulder capsule and age-related modifications of extracellular matrix are still poorly known [17, 23–26].

The aim of the present pilot study was to analyze the eventual age-related distribution of the elastic fibers in unaffected shoulder capsule and in patients with traumatic anterior and atraumatic multidirectional instability at first surgery. The hypothesis was that a different pattern of distribution of the elastic fibers might be associated with shoulder instability.

## 2. Materials and Methods

### 2.1. Patients and Specimen Collection

Biopsy specimens were obtained from the shoulder capsule of three groups of male patients from the senior authors practice between 2015 and 2017. All subjects gave informed written consent for the use of their specimens to the purpose of the present trial. The diagnosis of instability was made when there was a history of subluxation with spontaneous reduction or a history of dislocation requiring manual reduction, and patients had a positive anterior apprehension and relocation test (anterior instability) or anterior and posterior apprehension and positive sulcus test (multidirectional instability).

The patients were divided preoperatively into three different groups on the basis of history, clinical and instrumental (MRI/TAC) evaluation, and arthroscopic findings. The indication for arthroscopic shoulder stabilization was given independently from the study protocol.

**Group 1** consisted of 10 consecutive male patients with a history of traumatic shoulder dislocation/s and persistent clinical or radiographic evidence of anteroinferior shoulder instability (labral detachment, anterior labroligamentous and periosteal sleeve avulsion lesion [ALPSA]).

Inclusion criteria were at least 1 shoulder dislocation, traumatic onset before 6 months, and unidirectional instability with or without hyperlaxity (grades B2 or B3 according to Gerber and Nyffeler [18]. Exclusion criteria were multidirectional instability or evidence of bony glenoid defects (>25% as obtained on Bernageau radiographic profile or 3-dimensional computed tomography scans). The number of reported dislocations varied from 3 to 11 episodes (median value: 5). The average age of these patients was 28 years (ranging from 17 to 40).

All patients were treated by arthroscopic capsulolabral repair and refixation with sutures and anchors technique.

**Group 2** consisted of 10 consecutive patients with clinical symptoms and signs (lasting for at least 6 months) indicating instability in an inferior direction and at least one other direction (anterior or posterior), without history of trauma [21].

The hyperabduction test, or Gagey test, was conducted to evaluate passive abduction, which occurs within the glenohumeral joint and is controlled by the inferior glenohumeral ligament. In patients with instability, 85%, show a range of passive abductions of over 105 degrees versus 90 degrees in the contralateral shoulder, the former associated with lengthening and laxity of the inferior glenohumeral ligament [27]. The number of reported dislocations varied from 5 to 10 episodes (median value: 6). The average age of these patients was 30 years (ranging from 16 to 42).

Findings at arthroscopy and imaging reflected the dominant direction of instability. The most common arthroscopic finding was increased capsular volume based on redundant axillary recess without intra-articular associated lesions. All patients were treated by arthroscopic capsular plications for MDI.

**Group 3**: the control group consisted of samples from shoulder capsule of 10 male patients with no history of instability: 3 patients underwent arthroscopic repair of acute traumatic glenoid fracture and 7 underwent open reduction and osteosynthesis of proximal humeral fracture. The average age of these patients was 34 years (ranging from 16 to 45).

Only male patients were enrolled in this study in order to limit histological variations due to sex.

In addition to the group as a whole, specific subgroups were analyzed separately on the basis of the age of subjects: > 22 or < to 22 years. In particular, Groups 1A, 2A, and 3A included subjects with age < 22 years whereas Groups 1B, 2B, and 3B included subjects with age > 22 years. Age represents an important prognostic factor for redislocations [28, 29]. Some authors have defined the cut-off for increased risk of redislocation in the early third decade (20 years [27], 22 years, and 25 years [11]), whereas others have found different risks in patients aged less than 20 years (64%) versus greater than 40 years (6%) [30].

In our study, age was categorized into clusters of younger than 22 years and 22 years or older, based on the findings from a previous study by the senior authors, where it was shown that patients younger than 22 have a greater risk of redislocation after arthroscopic treatment of instability [29].

We excluded from the study all subjects affected by shoulder instability associated with voluntary dislocations, unequivocal diagnosis of genetic disorders (i.e., Marfan Syndrome and Ehler-Danlos Syndrome), and cervical or other neurological diseases predisposing to shoulder instability.

At the time of surgery, a capsular biopsy was harvested from the capsule in the interval between the middle and the inferior glenohumeral ligaments (Rouviere Foramen), in all patients of Groups 1 and 2 during the capsular shift procedure. The specimens were obtained by an arthroscopic biopsy punch about 1 cm lateral to the glenoid rim. The average size of the specimens used for analysis was 3 x 3 mm. Biopsy from the same region was taken from the control group subjects (Group 3) by arthroscopy in 3 cases and by open procedure in 7 cases. All the samples were analyzed by immunohistochemical and histomorphometric methods. The images were evaluated separately by two senior experts on soft tissue histology blinded to all clinical parameters. The independent evaluations were then discussed by the two observers to reach consensus and to obtain the final evaluation on each slide.

### 2.2. Histochemistry

For light microscopy, specimens were fixed by immersion in 4% paraformaldehyde in 0.1 M phosphate buffer, pH 7.4, at 4°C for 24 hours, decalcified in 4 N formic acid and sodium citrate for 72 hours, embedded in paraffin, cut into transverse and longitudinal sections (3 to 5 *μ*m thick), and stained with haematoxylin-eosin for assessment of the quality of the tissue, with specific methods for EFs (Weigert's resorcinol fuchsin and Verhoeff's iron hematoxylin) and with Van Gieson (for collagen fibers).

### 2.3. Immunohistochemistry

Sections were deparaffinized in xylene and rehydrated in graded ethanol. Intrinsic peroxidase activity was blocked by immersion in distilled water containing 3% hydrogen peroxidase for 6 min. Nonspecific binding was blocked with 3% normal goat serum in phosphate-buffered saline (PBS), pH 7.4, for 30 min at room temperature. Slides were then incubated overnight at 4°C with the primary antibodies. A monoclonal antielastin antibody (Sigma-Aldrich, Milan, Italy) was used at 1:5000 dilution. Rabbit and mouse immunoglobulins at the same dilutions as the primary antibodies were used as controls. The reactions were revealed using the DAKO LSAB + kit, HRP (Dako, Carpinteria, CA, USA). The analysis of immunolabeling of elastin was performed on two slides per specimen by evaluating five fields in each slide and defining three different grades: 1 low, 2 moderate, and 3 high immunolabeling. Staining was viewed and photographed with a Leica Microscope.

### 2.4. Histomorphometry

Computerized morphometric analysis of EFs was performed by using the Leica Q500 MC Image Analysis System (Leica Cambridge Ltd., Cambridge, England) on two slides per specimen stained with Verhoeff's iron hematoxylin. All morphometric steps, except the section of the first microscopic field, were automated. EFs were counted electronically and labelled with different colors to facilitate their identification. The software for mathematical morphology calculated the area fraction (Area%) occupied by EF (i.e., pixels of EFs/256”pixels); five fields in each slide were studied.

#### 2.4.1. Statistical Analysis

Data were expressed as mean +- SD of the mean, and the statistical evaluation was carried out by ANOVA test. A value of p< 0.05 was considered significant. All statistical analysis was performed with SPSS, version 19.0 (IBM, Armonk, New York).

## 3. Results

**Figure 1:**
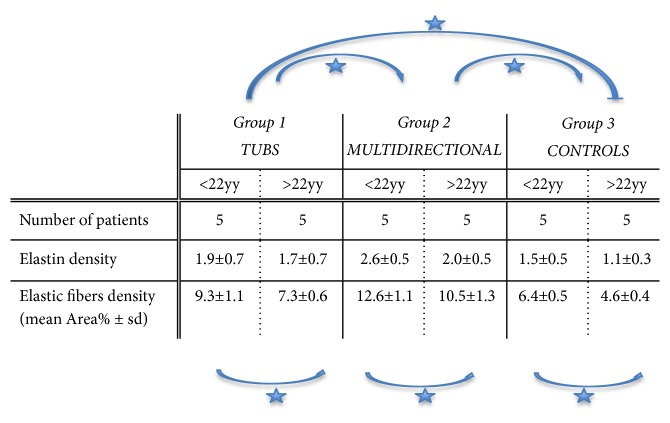
Number of patients, immunohistochemical and histomorphometric data of specimens from patients affected with traumatic instability (Group 1), multidirectional instability (Group 2), and control subjects (Group 3). All intragroup differences (<22 yy vs >22 yy) were statistically significant (see arrows with asterisks). All intergroup differences (Group 1 vs Group 2, Group 2 vs Group 3, and Group 1 vs Group 3) were statistically significant (see arrows with asterisks).

**Figure 2:**
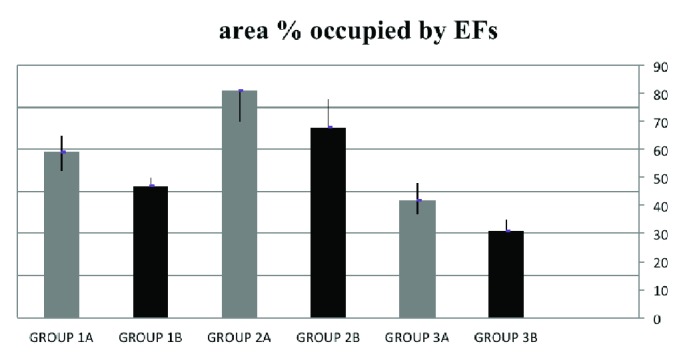
Histomorphometric data of EFs distribution (mean area % occupied by EFs ± SD) from patients with shoulder instability (Groups 1 and 2) and control subjects (Group 3). Subgroup A: patients < 22 yy old; Subgroup B: patients >22 yy old.

**Figure 3:**
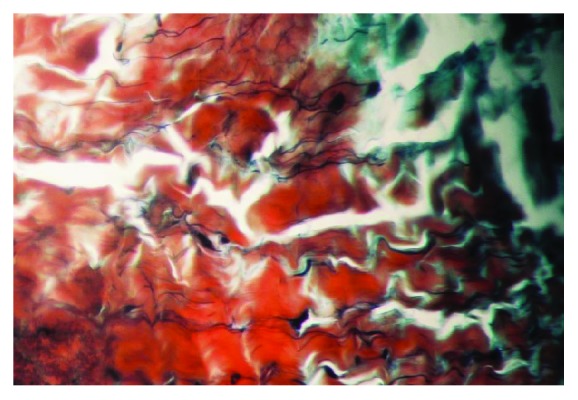
Shoulder articular capsule of an 18-year-old patient affected with MDI. Many elastic fibers are homogeneously distributed and arranged in bundles with a course parallel to collagen fibers, showing a crimp feature (Verhoeff's iron hematoxylin, staining, and original magnification, x 400; elastic fibers are stained in violet or purple, whereas collagen is in red).

**Figure 4:**
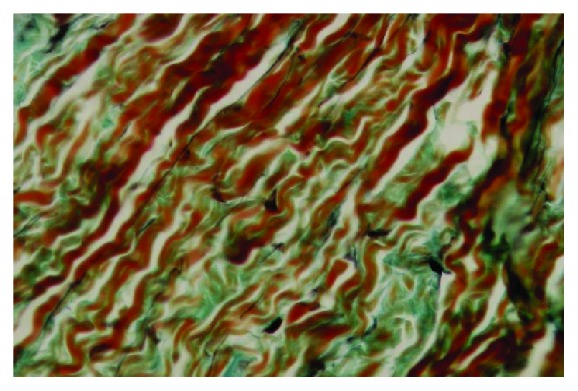
Shoulder articular capsule of a 33-year-old patient affected with MDI. Many elastic fibers maintain the normal orientation in the different layer but are reduced in number in comparison with patients of the same group with age < 22 yy (Verhoeff's iron hematoxylin staining, and original magnification x 400; elastic fibers are stained in violet or purple, whereas collagen is in red).

**Figure 5:**
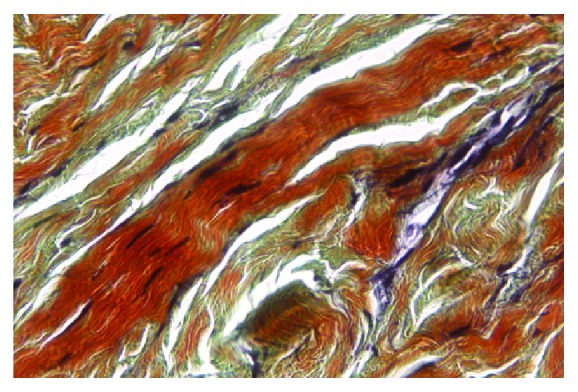
Shoulder articular capsule from a subject 40 years old of control group. Very few elastic fibers are detectable; their number is dramatically reduced in comparison with the patients of the other groups (Verhoeff's iron hematoxylin staining, original magnification x 400; elastic fibers are stained in violet or purple, whereas collagen is in red).

Data of the specimens from the patients with shoulder instability and control group were summarised in Figures 1 and 2. The topographic distribution of the EFs was similar, but both the elastin density and the percentage of area covered by EFs were higher in younger subjects (Groups 1A, 2A, and 3A) in comparison to older subjects (Groups 1B, 2B, and 3B). These differences were in all cases statistically significant (p<0.05). The elastin density and the percentage of area covered by EFs were higher in specimens of Group 2 (Multidirectional instability) in comparison to the other Groups (p<0.001), and this difference was statistically significant, as was the difference between Group 1 and Group 3 (p<0.001). No differences in the cellularity and vascularisation were observed between the specimens from different groups of subjects. EFs stained a bright violet or purple were flame-shaped and varied in length. Numerous EFs were homogeneously distributed in all the specimens analyzed. They were arranged in bundles with a preferential orientation (Figures 3, 4, and 5), as previously observed in human and rabbit articular capsules of the knee [25].

## 4. Discussion

The main finding of the present pilot study is that there is a significant difference in terms of elastin and elastic fibers density among patients younger or older than 22, especially in case of multidirectional shoulder instability, where the highest density of both elastin and EFs is documented.

Very few studies analyzed the biochemical composition and the histological structure of human normal and pathological shoulder capsule. Kaltsas et al. identified collagen types I,III, and V in shoulder capsule by gel electrophoresis, but no further biochemical and histological characterization was reported [13]. Ticker et al. showed that the overall biochemical composition of the inferior glenohumeral ligament in specimens from subjects with a mean age of 60 years was similar to the composition of other ligaments [31]. Hirakawa et al. reported the presence of abundant immature collagen fibers (increased cysteine content) in shoulder capsule from loose shoulders compared to controls [24]. Mc Farland et al. documented the histologic changes that occur in the capsule of patients with traumatic instability of the shoulder as a denuded synovial layer, subsynovial edema, increased cellularity, and increased vascularity [11]. Rodeo et al. analyzed collagen cross-links, collagen fibril diameter and density, amino acid composition, and elastic fibers in shoulder capsule and skin in four patient groups: (1) unidirectional anterior instability; (2) multidirectional instability/primary surgery; (3) multidirectional instability/revision surgery; and (4) no history of instability. The age-related changes were not considered. They did not find significant differences between the unidirectionally and multidirectionally unstable capsule, but samples from multidirectional instability/revision surgery subjects contained significantly more reducible cross-links, smaller-diameter collagen fibrils, decreased collagen fibril density, and an increased density of elastin staining. The authors showed that collagen fibrils could play a major role in shoulder instability and hypothesized that repeated capsular deformation in patients with shoulder instability may provide the stimulus for adaptive changes that increase the strength of the capsule in an attempt to diminish instability. Thus they interpreted the differences in EFs distribution in shoulder capsule as a consequence rather than as predisposing factor of shoulder instability [20].

To the best of our knowledge, no previous studies have measured the age-related distribution of EFs in normal and unstable shoulder capsule. The present study attempted to elucidate some age-related histological aspects of the capsule that may contribute to capsular laxity and predispose to first shoulder dislocation and recurrence. We decided to consider only male subjects and subjects under and over the age of 22 years because male sex and age under 22 years at time of surgery have been found as significant risk factors for recurrence [9].

Our observations showed the presence of numerous EFs in shoulder capsule of young and adult subjects. The topographic distribution of the EFs was similar, but their number (elastin density and percentage of area covered by EFs) was significantly higher in younger subjects in comparison to older subjects (age > 22y). Furthermore, shoulder capsule from patients with shoulder instability had higher density of EFs compared with normal capsule, particularly in the samples taken from patients affected with multidirectional instability.

The mechanical properties of articular capsule and ligaments are due to their extracellular matrix that is mainly composed of water, glycosaminoglycans, collagen, and elastic fibers [16]. EFs consist of an amorphous core of elastin and closely associated microfibrils that are composed of fibrillin and microfibrillar associated glycoproteins. Elastin, the major component of mature EFs, should provide the elastomeric properties required. Microfibrils, which constitute the so-named oxytalan fibers, probably do not elongate much under mechanical stress. They are more abundant in location where resistance to mechanical stress is required, such as periodontium and osteoperiosteal junction. To date, however, the functional role of EFs in the joint capsule has not been clarified yet, even though they could have a role in the rapid recovery of the initial length after deformation. Previous studies demonstrated the topographic distribution and the mechanical role of EFs in several musculoskeletal tissues. Some of us described the age-related distribution of the EFs in the rabbit knee and in the human epiphyseal region and made hypotheses on the possible role of these fibers in maintaining joint stability and in affecting the growth process of long bones [18, 21, 32]. We found that the type and the quantity of fibers varied according to the age of the subjects in various skeletal tissues, including articular capsule of the knee.

Some studies have suggested the involvement of these fibers in several genetic and acquired pathological conditions, including some affecting the musculo-skeletal system [28, 29, 33]. In previous studies we observed that in human and bovine Marfan syndrome the structural abnormalities of EFs in articular capsule could make the capsule functionally incompetent to resist normal stress, predisposing to joint laxity and dislocations [27, 34, 35].

Data of the present study confirmed also in human articular capsule of the shoulder the presence of an age-related distribution of EFs, and showed a greater amount of these fibers in unstable shoulder. These data confirm the observations of Rodeo et al. about increased density of elastin staining in samples from patients affected by multidirectional instability [20].

This study however presents some weakness related to the small number of subjects included, the limited area of the capsule sampled, the histological analyses limited to EFs, and the fact that just the 22-year-old cut-off was considered. In particular, the biopsy location may affect the histological results: we took biopsy samples from the Rouviere Foramen, close to the middle glenohumeral ligament, 1 cm lateral to the capsulolabral edge. Future studies should sample different areas of the capsule in order to verify whether the extracellular matrix composition changes inherently. We focused the analyses on the EFs. However, the mechanical properties of ligaments and articular capsule are also due to other extracellular matrix components such as glycosaminoglycans/water and collagen fibers, which will be age-related analysis in further studies [31]. The choice of the cut-off at the age of 22 was performed based on the findings coming from clinical trials previously published: anyway we cannot exclude the fact that other age-related differences might exist and the preliminary results of the present pilot study could be used to design a bigger trial to eventually detect those difference. Unfortunately, due to ethical reasons, it was not possible to include more biopsies in this study and our sample size did not allow for further statistically sounding analysis. In light of these findings, it is difficult to understand all the factors affecting capsular strength and to know if the extracellular matrix composition is the cause rather than consequence of shoulder instability.

## 5. Conclusion

Based on the findings of this pilot study, a greater elastin density and EFs distribution in capsular biopsies of younger subjects with unstable shoulders compared to healthy controls may represent an important factor in predisposing the joint to first dislocation and recurrence, particularly in the subgroup of patients with multidirectional shoulder instability.

## Data Availability

The data used to support the findings of this study are available from the corresponding author upon request.
